# PARP Inhibitors in the Neoadjuvant Setting; A Comprehensive Overview of the Rationale for their Use, Past and Ongoing Clinical Trials

**DOI:** 10.1007/s11912-025-01669-z

**Published:** 2025-04-07

**Authors:** Minatoullah Habaka, Gordon R. Daly, Deborah Shinyanbola, Mohammad Alabdulrahman, Jason McGrath, Gavin P. Dowling, Cian Hehir, Helen Ye Rim Huang, Arnold D. K. Hill, Damir Varešlija, Leonie S. Young

**Affiliations:** 1 Department of Surgery, RCSI University of Medicine and Health Sciences, Dublin, Ireland; 2 Department of Surgery, Beaumont Hospital, Dublin, Ireland; 3 School of Pharmacy and Biomolecular Sciences, RCSI University of Medicine and Health Sciences, Dublin, Ireland; 4 Beaumont RCSI Cancer Centre, Beaumont Hospital, Dublin, Ireland

**Keywords:** Neoadjuvant therapy, PARP inhibitors, Synthetic lethality, HRR, HRD, BRCA1/2, Breast cancer, Ovarian cancer, Prostate cancer

## Abstract

**Purposeof Review:**

Poly (ADP-ribose) polymerases (PARPs) are enzymes essential for detecting and repairing DNA damage through poly-ADP-ribosylation. In cancer, cells with deficiencies in homologous recombination repair mechanisms often become more dependent on PARP-mediated repair mechanisms to effectively repair dsDNA breaks. As such, PARP inhibitors (PARPis) were introduced into clinical practice, serving as a key targeted therapy option through synthetic lethality in the treatment of cancers with homologous recombination repair deficiency (HRD). Though PARPis are currently approved in the adjuvant setting for several cancer types such as ovarian, breast, prostate and pancreatic cancer, their potential role in the neoadjuvant setting remains under investigation. This review outlines the rationale for using PARPi in the neoadjuvant setting and evaluates findings from early and ongoing clinical trials.

**Recent Findings:**

Our analysis indicates that numerous studies have explored PARPi as a neoadjuvant treatment for HRD-related cancers. The majority of neoadjuvant PARPi trials have been performed in breast and ovarian cancer, while phase II/III evidence supporting efficacy in prostate and pancreatic cancers remains limited.

**Summary:**

Studies are investigating PARPi in the neoadjuvant setting of HRD-related cancers. Future research should prioritize combination strategies with immune checkpoint inhibitors and expand outcome measures to include patient satisfaction and quality-of-life metrics.

## Introduction

Poly (ADP-ribose) polymerase (PARP) are a family of enzymes that play an integral role in various cellular processes, most notably in detecting and repairing DNA damage [1]. Under genotoxic stress, single-stranded DNA (ssDNA) breaks lead to depletion of nicotinamide adenine dinucleotide (NAD) and subsequent activation of PARP enzymes, particularly PARP1 [2]. Once activated, PARP1 is recruited to sites of DNA damage via its zinc-finger domains (Zn1 and Zn2) [2]. The interaction between these domains and the nucleotide bases induces a structural rearrangement in Zn1/Zn2, resulting in allosteric activation [2, 3].This process involves cleaving NAD and catalyzing the addition of poly(ADP-ribose) (PAR) groups to both DNA and the enzyme itself, a modification known as poly-ADP-ribosylation (PARylation) [2, 3]. This modified PARP1 subsequently coordinates with other enzymes within the base excision repair (BER) complex to facilitate DNA repair [2]. PARP inhibitors (PARPis) can disrupt this process by competitively binding to the catalytic domain of PARP, thereby preventing NAD interaction and inhibiting PARylation [2]. In addition to PARPis competing for the catalytic domain, they also trap PARP enzymes attached to DNA by binding to the PARP-DNA complex [4]. By trapping the complex, the replication fork collapses and ultimately blocks DNA repair mechanisms [4]. With both mechanisms, the accumulation of unrepaired ssDNA results in double-stranded DNA (dsDNA) breaks, which are repaired by homologous recombination repair (HRR) [2].

**Fig. 1:**
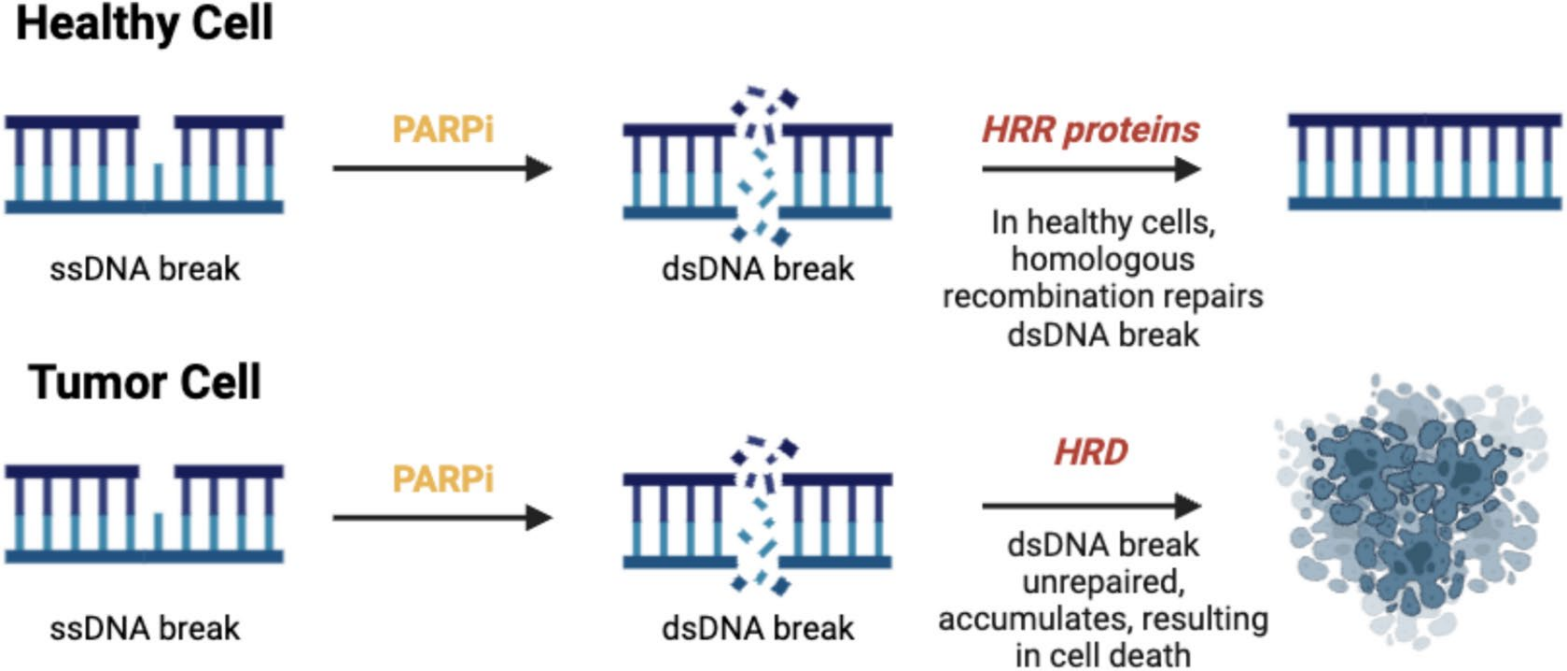
Schematic representation showing a normal cell with a functional HRR pathway effectively repairing dsDNA breaks following PARP inhibition of ssDNA breaks versus cells with HRD unable to repair dsDNA breaks, resulting in accumulation of DNA damage and cell death

In the context of cancer with homologous recombination repair deficiency (HRD), cells often have germline or somatic mutations in genes that code for HRR proteins required for the repair pathway [5, 6]. Cells with this deficiency cannot effectively repair dsDNA breaks via HRR and are more reliant on PARP-mediated repair mechanisms [1, 5–7]. This dependency makes PARP a strategic target for cancer therapies as inhibiting PARP can lead to the accumulation of unrepaired DNA damage in cancer cells, ultimately resulting in cell death [7]. This approach is termed “synthetic lethality” and has underscored the efficacy of PARPis as a well-established therapeutic strategy in the adjuvant treatment of breast, prostate, pancreatic, ovarian and other gynaecological malignancies (Fig. 1) [6–8]. More recently, the potential role of PARPis in the neoadjuvant setting is being explored. This review will summarise; the rationale for using PARPis in the neoadjuvant setting, ongoing clinical trials assessing the benefit of neoadjuvant PARPi treatment, and the future directions of the use of neoadjuvant PARPis.

### Homologous Recombination Repair Mechanisms

HRR is a high-fidelity mechanism that repairs dsDNA breaks during the S to G2 phases of the cell cycle and is critical after exposure to cytotoxic agents or ionizing radiation [9–11]. Initially, the MRN complex detects dsDNA breaks and BLM and EXO1 for DNA resection [9, 11, 12]. The resulting ssDNA overhangs are coated with RPA, which prevents excessive resection [9, 11, 12]. ATR then binds to the RPA-coated ssDNA, activating the ATR-Chk1 checkpoint to halt the cell cycle and protect replication forks, with ATM kinase activating downstream targets like BRCA1 protein [9, 11, 12]. Following cell cycle arrest, RAD51 replaces RPA on the ssDNA, forming presynaptic filaments with the help of BRCA2 and PALB2 [9, 12, 13]. The RAD51 nucleofilament searches for and invades the homologous dsDNA on the sister chromatid, creating a displacement loop [11, 13]. DNA synthesis and ligation occur using the intact chromatid as a template, and the process concludes with the resolution of Holliday junctions by the BTRR dissolvasome, thus completing HRR [9, 10, 13]. 13–17% of breast, ovarian, and pancreatic cancers with mutations in genes within the HRR pathway present with an HRD phenotype [10]. Notable genes associated with HRD include *BRCA1, BRCA2, ATM, BARD1, PALB2, RAD51C, RAD51D,* with variable patterns of gene-specific mutations observed across different cancer types [10]. For example, mutations in *BRCA1/2*, *ATM* and *PALB2* are most associated with breast cancer; mutations in *ATM* and *BRCA2* are commonly associated with prostate cancer; mutations in *BRCA1/2* are commonly associated with ovarian cancer [14]. Table 1 summarises genes defects that are associated with HRD and their corresponding cancer risk.

**Table 1:** Common gene defects in HRR pathway and associated cancer risk according to the ESMO Clinical Practice Guideline [14]

Mutation	Associated Cancer with Greatest Risk (Lifetime Risk of HBOC Associated PV’s (%))	Other Associated Cancer(s) (Lifetime Risk of HBOC- Associated PV’s (%))
*ATM*	Breast (25–30) Prostate (30)	Ovarian (< 5) Pancreatic (< 5)
*BARD1*	Breast (20)	-
*BRCA1*	Breast (> 60) Ovarian (40–60)	Pancreatic (< 5)
*BRCA2*	Breast (> 60)	Ovarian Cancer (15–30) Pancreatic Cancer (5) Prostate Cancer (33)
*BRIP1*	Ovarian (5–10)	-
*PALB2*	Breast (40–60)	Ovarian (3–5) Pancreatic (2–3)
*RAD51C*	Breast (20)	Ovarian (10)
*RAD51D*	Breast (10) Ovarian (10)	-

Abbreviations: HBOC = Hereditary breast and ovarian cancer, PV = pathogenic variant

HRD detection has significant therapeutic implications, specifically by increasing sensitivity to PARPis [15]. Therefore, fast, accurate and cost-effective identification of HRD is an area of increasing importance. Currently, genetic testing, genomic assays and functional assays are methods that can measure HR. Genomic scars HRD tumours are detectable abnormalities as a result of genomic instability [16, 17]. The primary method for detecting these scars is measuring somatic copy number alterations (SCNAs) and additional indicators of chromosomal rearrangement such as large-scale transitions, telomeric allelic imbalance and loss of heterozygosity [16, 18–22]. Mutational signatures of HRD are characteristic patterns of DNA alterations resulting from various processes that damage and repair a cell's genome [16]. These signatures are typically measured using whole genome sequencing (WGS) or whole exome sequencing (WES) [16, 23, 24]. The Foundation Medicine CDxBRCA LOH and Myriad Genetics myChoice are two FDA-approved assays that detect gene mutations [16]. Alternatively, functional assays aim to directly test HRR proficiency with one method measuring the amount of nuclear RAD51, which is DNA recombinase involved in the HRR pathway, as previously mentioned [16]. However, measurement of RAD51, amongst other clinical assays have proved thus far limited due to the time or expense associated with their performance [16, 25].

### PARPis in the Adjuvant Setting for HRD-associated Cancers

The FDA has approved the following PARPis in the adjuvant setting for cancers with HRD: olaparib for ovarian, breast, prostate and pancreatic cancers [26]; talazoparib for breast and prostate cancer [27]; rucaparib for ovarian and prostate cancer [28]; niraparib for ovarian cancer [26, 27, 29].

PARPis are well-established in the adjuvant treatment of gBRCAm breast cancer in both high risk early and metastatic settings [30]. Olaparib is indicated for patients with early high-risk, or metastatic human epidermal growth receptor-2 (HER2)-negative, germline *BRCA* mutated (*gBRCAm*) disease [26]. In addition, it can be considered as a second or third line agent after chemotherapy or endocrine therapy if estrogen receptor (ER)-positive regardless of menopausal status. Talazoparib is approved for single agent treatment of *gBRCAm* HER2-negative locally advanced or metastatic breast cancer [27]. Both drugs initially gained licensing in the advanced setting following the OlympiAD and EMBRACA trials [31–33]. These phase III randomised control trials (RCTs) reported significantly improved progression-free survival (PFS) in the PARPi cohorts compared to treatment of physician’s choice (TPC) respectively [31, 33]. The licensing of olaparib was extended to *gBRCA1/2 m,* high-risk, HER2-negative early breast cancer following the results of the phase III OlympiA trial which observed a 4-year OS benefit in patients treated with olaparib versus placebo [34].

In the treatment of different forms of ovarian cancer, olaparib, rucaparib, and niraparib are currently approved as adjuvant therapies [35, 36]. All three of these PARPis are approved for maintenance treatment in patients with advanced or recurrent epithelial ovarian, fallopian tube, or primary peritoneal cancer, who are in complete or partial response to platinum-based chemotherapy [26]. Additionally, olaparib is also indicated for patients with gBRCAm or hormone receptor-positive (HR +) disease, in the first-line setting and recurrent disease, including patients with complete or partial response to platinum-based chemotherapy [26]. The benefit of PARPi treatment in this cohort was highlighted by several clinical trials including the SOLO1 study, which reported olaparib to be associated with a significantly improved PFS of 56.0 months versus 13.8 months with placebo [37, 38]. Similarly, the phase III PAOLA-1/ENGOT-ov25 trial demonstrated a 5-year PFS of 46.1% with a combination of olaparib and bevacizumab versus 19.2% with placebo [39]. Additionally, olaparib significantly increased time to requirement of subsequent therapy compared to control (hazard ratio, 0.59; 95% CI, 0.49 to 0.71) [40]. The ARIEL3 trial, a phase III trial, investigated the use of rucaparib in platinum-sensitive ovarian cancer and found a PFS of 16.6 months in the gBRCAm group receiving rucaparib and 13.6 months in the gBRCA wildtype, HRD group, compared to 5.4 months in the placebo group [41]. The PRIME phase III trial demonstrated the benefit of niraparib patients with a broad range of mutational statuses [42]. Median PFS was 24.8 months with niraparib versus 8.3 months with placebo in the overall population with a follow-up period of 27.5 months [42]. For patients with gBRCAm, PFS was not reached with niraparib compared to 10.8 months with placebo [42]. In non-BRCAm patients, PFS was 19.3 months with niraparib versus 8.3 months with placebo [42]. Among patients with HRD (including non-BRCAm), PFS was not reached with niraparib versus 11.0 months with placebo. In patients with proficient HRR, PFS was 16.6 months with niraparib versus 5.5 months with placebo [42]. The PRIMA phase III study compared niraparib as maintenance therapy to placebo in HRD ovarian cancer following platinum chemotherapy [43]. PFS was longer in the niraparib group, with 13.8 months versus 8.2 months in the placebo group [43].

Prostate cancer is typically treated with surgery, radiotherapy, and androgen deprivation therapy (ADT), with chemotherapy reserved for advanced or metastatic cases [44]. Neoadjuvant chemotherapy and the use of PARPis is investigated to treat aggressive cancers and those with HRD. In metastatic castration-resistant prostate cancer (mCRPC), the FDA has approved four PARPis: olaparib, talazoparib, rucaparib, and niraparib [45, 46]. Olaparib is indicated in gBRCAm and HRD mCRPC previously treated with androgen receptor and biosynthesis inhibitors; enzalutamide or abiraterone [26]. This treatment strategy gained favour following the PROfound trial which assessed olaparib compared to the physician's choice of enzalutamide or abiraterone and reported PFS of 7.4 months with olaparib versus 3.6 months with placebo [47]. Furthermore, in the phase III PROpel study, combination olaparib and abiraterone increased PFS to 24.8 months compared to placebo and abiraterone with a PFS of 16.6 months, in mCRPC [48]. Additionally, talazoparib is used with enzalutamide in mCRPC with gene defects in the HRR pathway [27]. The TALAPRO-1 phase II study investigated talazoparib which showed radiographic PFS (rPFS) of 8.2 months for patients with gBRCAm and 3.5 months for patients with ATM mutation [49]. Lastly, the Phase II TRITON2 study led to the approval of rucaparib for mCRPC previously treated with androgen receptor-directed therapy and taxane-based chemotherapy [28]. This clinical trial identified higher efficacy of rucaparib based on the PSA50 response rates (defined as a ≥ 50% decrease in prostate-specific antigen (PSA) from baseline) as follows: 53% in the BRCA subgroup, 55% in the PALB2 subgroup, 3.4% in the ATM subgroup, 6.7% in the CDK12 subgroup, 14% in the CHEK2 subgroup, and 23% in other subgroups [50]. In the phase III MAGNITUDE trial, niraparib combined with abiraterone acetate and prednisone demonstrated a PFS of 19.5 months compared to 10.9 months in the control group receiving abiraterone acetate and prednisone [51].

In pancreatic cancer, olaparib is approved for the treatment of gBRCAm metastatic pancreatic adenocarcinoma that has not progressed after at least 16 weeks of a first-line platinum-based chemotherapy [26]. This licensing was approved following the phase III POLO trial that assessed olaparib in patients with gBRCAm and metastatic pancreatic cancer and disease that had not progressed during platinum-based chemotherapy. POLO reported a PFS of 7.4 months with olaparib vs. 3.8 months with placebo [52].

### Overview of PARPis in Neoadjuvant Setting

**Fig. 2:**
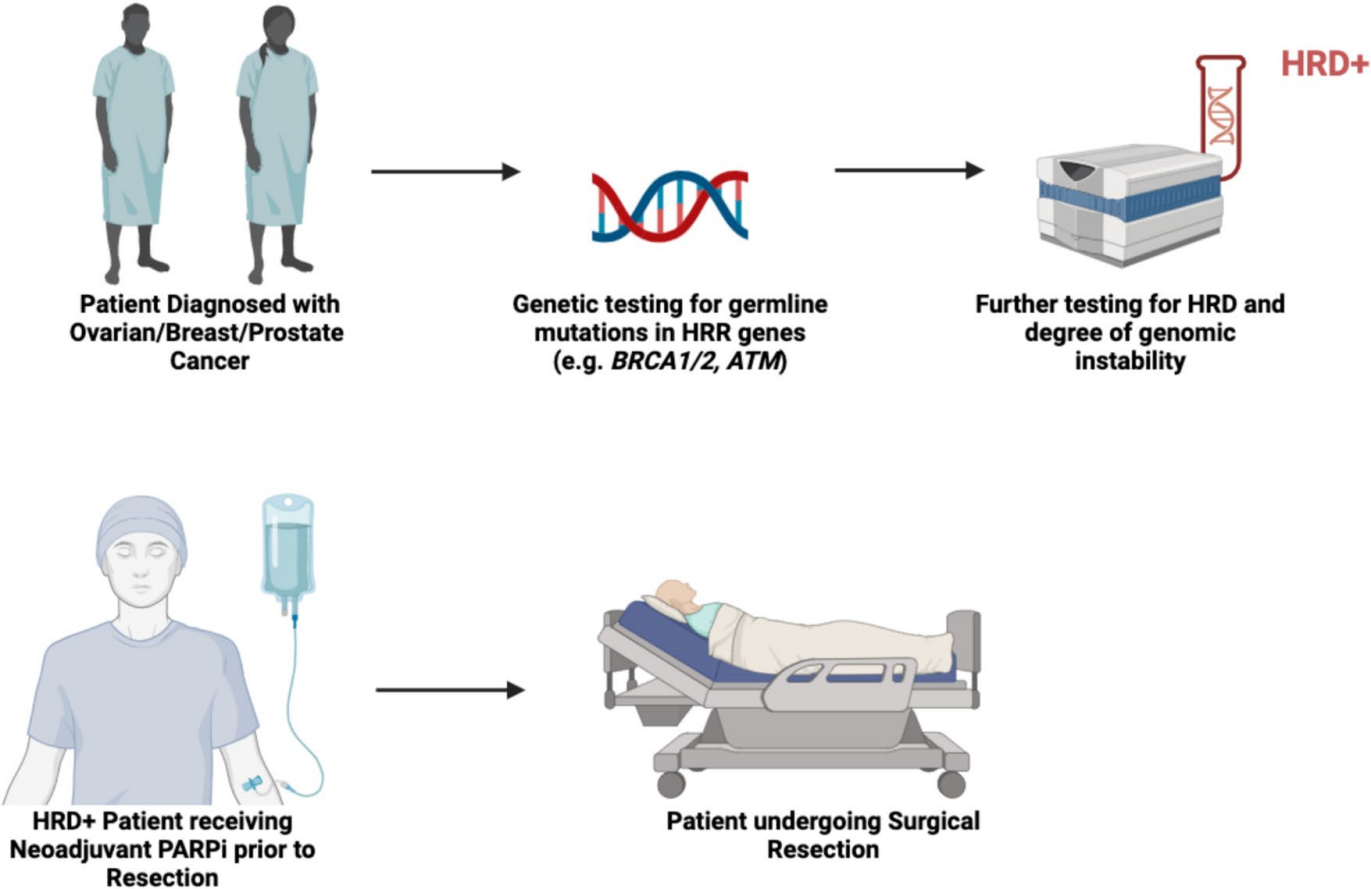
Overview of neoadjuvant therapy prior to surgical resection

Neoadjuvant therapy refers to chemotherapy, radiotherapy or endocrine therapy that is administered prior to local resection, primarily serving the purpose of downstaging tumours for manageable surgical resection and providing information on treatment response as well as prognosis [53]. This approach is commonly used in clinical practice across many cancer types such as breast, prostate, ovarian, pancreatic, lung and colorectal cancer [54]. Most prominently, the benefits of neoadjuvant chemotherapy (NAC) in breast cancer, regardless of hormone-receptor status, have been well-established in the literature [55, 56]. In advanced ovarian cancer, NAC followed by interval debulking surgery (IDS) and adjuvant chemotherapy showed slightly improved survival outcomes, reducing the risk of serious surgery-related adverse events, postoperative mortality and the need for stoma formation [57–59]. In contrast to chemotherapy alone, the addition of neoadjuvant immunotherapy to surgery and adjuvant immunotherapy has shown promising efficacy by enhancing immune responses such as presentation of neoantigens, enhanced cell death, and inflammatory responses [57]. PARPis offer a personalized targeted approach in the treatment of HRD-positive breast, ovarian and prostate cancer, and their potential benefit in the neoadjuvant setting is currently being elucidated in a number of ongoing clinical trials. The neoadjuvant treatment strategy using PARPis is summarised in Fig. 2.

### Past Clinical Trials on Neoadjuvant PARPis in Breast Cancer

Several clinical trials have explored the safety and efficacy of PARPis in the neoadjuvant setting for breast cancer with variances in results across each study in terms of results (Table 2). I-SPY2, a phase II study, investigated olaparib in combination with durvalumab in patients with stage II/III hormone receptor-positive/HER2-negative breast cancer [19]. The trial demonstrated a significant increase in pathological complete response (pCR) rates compared to standard paclitaxel treatment (64% vs. 22%, p < 0.001) [19]. However, the study also reported higher rates of grade 3 or higher adverse events (AEs) in the intervention group compared to the control (56% vs. 34%), with immune-related AEs occurring in 27.4% of the intervention group versus 2% in the control group [19]. However, similar to the adjuvant setting, neoadjuvant PARPi treatment was associated with equivocal or reduced AEs than chemotherapy in the GeparOla trial which evaluated the addition of olaparib to paclitaxel compared to the combination of paclitaxel and carboplatin. Here, whilst the PARPi cohort had a significantly higher pCR rate (55.1% vs. 48.6%, p = 0.04), only 46.4% of patients in the intervention arm experienced grade 3 or higher hematologic toxicities compared to 78.4% in the control group, with 13% of the intervention group experiencing other AEs compared to 54.1% in the control [18]. These findings suggested that the addition of targeted PARPi therapy was les​s toxic than dual chemotherapy in this cohort [18].

**Table 2:** Summary of completed or terminated trials investigating PARPi in neoadjuvant treatment of breast cancer (BC)

Study (trial number)	Year	Phase	Condition	Inclusion Criteria	Neoadjuvant treatment	Treatment Regimen	Control	Control Regimen	pCR	Safety and Toxicity
I-SPY2 Trial (NCT01042379)	2021	II	Stage II/III BC	HR + /HER2-ve	PD-L1 inhibitor durvalumab + PARP inhibitor olaparib in addition to paclitaxel (n = 73)	Weekly	Paclitaxel (n = 299)	Paclitaxel 80 mg/m2 IV during 12 weekly treatment cycles; Doxorubicin 60 mg/m2 IV after completion of 12 weekly treatment cycles and prior to surgery for weeks 13–16; Cyclophosphamide 600 mg/m2 IV after completion of the 12 weekly treatment cycles and prior to surgery for weeks 13–16	64% (intervention) vs 22% (control)	≥ Grade 3 AEs: 34% control and 56% intervention; Immune-related AEs: 2% control and 27.4% intervention
PARTNER (NCT03150576)	2016	II/III	TNBC / gBRCA BC	ER-ve, and HER2-ve, any PR status OR g*BRCA*, HER-ve, any HR status	PARPi olaparib + paclitaxel/carboplatin (n = 327)	12 weeks: olaparib PO twice daily (12 h apart); paclitaxel IV 80 mg/m2 in 0.9% sodium chloride 500 ml over 60 min on days 1, 8 & 15, every 3 weeks for 4 cycles; carboplatin I.V. AUC5 in 5% dextrose 500 ml over 30–60 min on day 1 every 3 weeks for 4 cycles	Paclitaxel + carboplatin (n = 320)	12 weeks: paclitaxel IV 80 mg/m2 in 0.9% sodium chloride 500 ml given over 60 min on days 1, 8 & 15, every 3 weeks for 4 cycles; carboplatin IV AUC5 in 5% dextrose 500 ml over 30–60 min on day 1 every 3 weeks for 4 cycles	51% (intervention) vs 52% (control)	Stopped treatment due to toxicity: 14.1% control and 16.6% intervention; ≥ 3 AEs: 58.7% control and 64.2% intervention
GeparOla (NCT02789332)	2020	II	HER2-ve/TNBC	HER2-ve OR HRD OR TNBC	PARPi olaparib + paclitaxel (n = 69)	12 weeks: olaparib PO 100 mg twice daily; paclitaxel 80 mg/m^2^ IV weekly	Paclitaxel + carboplatinum (n = 37)	12 weeks of paclitaxel 80 mg/m^2^ IV weekly in combination; carboplatin AUC 2 IV weekly	55.1% (intervention) vs 48.6% (control)	≥ Grade 3 hematologic toxicities: 78.4% control and 46.4% intervention; AEs: 54.1% control and 13.0% intervention
BRIGHTNESS Trial (NCT02032277)	2018	III	Stage II-III TNBC	TNBC	PARPi veliparib + paclitaxel + carboplatin (n = 316)		Paclitaxel + carboplatin (n = 160); Paclitaxel (n = 158)		53% (intervention) vs 58% (paclitaxel + carboplatin) vs 31% (paclitaxel)	≥ Grade 3 AEs: neutropenia 56%, anaemia 29%, thrombocytopenia 12%, febrile neutropenia 15%
Neoadjuvant talazoparib (NCT03499353)	2021	II	Early-stage gBRCATNBC	g*BRCA*1/2 and HER2-ve	Talazoparib (n = 48)	24 weeks: PO 1 mg/day			45.8% (not meeting the prespecified threshold)	≥ Grade 3 AEs: fatigue 77%, nausea 63.9%, alopecia 57.4%; ≥ 3 anaemia 39.3%, neutropenia 9.8%
NCT03329937	2021	I	Localized BC	*BRCA*1/2 HER2-ve	Niraparib (n = 21)	2 28 day cycles: 100 mg PO at starting dose of 200 mg PO			38.10%	≥ Grade 3 AEs: anaemia 23.81%, neutropenia 9.52%, thrombocytopenia 4.76%
PETREMAC (NCT02624973)	2020	II	TNBC	primary TNBC (tumor size > 2 cm)	Olaparib (n = 32)	Olaparib 300 mg b.i.d. for up to 10 weeks			No pCR was observed with olaparib monotherapy or when followed by low-dose carboplatin prompting protocol amendments	one patient experienced > grade 2 toxicity (fatigue) requiring dose reduction

Abbreviations: TNBC = triple negative breast cancer, pCR = pathological complete response, ITT = intention to treat, AE = Adverse event, BC = breast cancer, HR +  = hormone receptor positive, HER2-ve = human epidermal growth factor receptor 2 negative, PD-L1 = programmed cell death ligand 1. PARPi = poly(ADP-ribose) polymerase inhibitor, gBRCAm = germline BRCA mutated, HRD = homologous recombination deficiency, PO = oral, IV = intravenous, AUC = area under curve, PFS = progression-free survival, b.i.d. = twice a day.

Despite the promising findings of I-SPY2 and GeparOla, other trials have provided contrasting results. The BRIGHTNESS trial assessed the combination of veliparib with paclitaxel and carboplatin; reporting pCR rates of 53% with veliparib-carboplatin combination compared to 58% with paclitaxel-carboplatin combination therapy, and 31% with paclitaxel alone (*p* < 0.05 for both comparisons) [60]. As commonly observed in previous trials, neutropenia was the most common grade 3 or higher adverse event (AE), affecting 57% of the intervention group, 53% of the paclitaxel and carboplatin group, and 3% of the paclitaxel group [60]. Furthermore, on median follow-up of 4.5 years, the addition of veliparib to carboplatin and paclitaxel added no significant benefit to event-free survival in this study [61]. Importantly, BRIGHTNESS included patients with TNBC irrespective of HRR status. These results underscore the importance of patient selection and the identification of HRD positivity prior to administration of PARPis [61]. This tailored approach avoids unnecessarily exposing patients to drug toxicity in cases where PARPi treatment will not improve disease outcomes.

The PARTNER trial, which evaluated olaparib with paclitaxel and carboplatin, demonstrated comparable pCR rates to the control group receiving paclitaxel and carboplatin alone (51% vs 52%, HR 1.02, 95% CI 0.75–1.39) [62]. Notably, 16.6% of patients in the intervention arm discontinued treatment due to toxicity, compared to 14.1% in the control arm, with grade 3 or higher AEs occurring in 64.2% of the intervention group versus 58.7% in the control​ (62). Additionally, in the NEOTALA trial, neoadjuvant talazoparib monotherapy achieved a pCR rate of 45.8% (95% CI, 32.0%−60.6%) and 49.2% (95% CI, 36.7%−61.6%) in the evaluable and intention-to-treat [63] population, respectively [33]. AEs associated with talazoparib included fatigue (77%), nausea (63.9%), and alopecia (57.4%), with grade 3 or higher anaemia occurring in 39.3% and neutropenia in 9.8% of patients [32]. In the phase I study of niraparib (NCT03329937) in neoadjuvant therapy of localized BC, the trial reported a pCR rate of 38.10% (95% CI: 18.1) [64]. Regarding safety, notable grade 3 or higher AEs included anaemia in 23.81% of participants, neutropenia in 9.52%, and thrombocytopenia in 4.76% [64].

These trials suggest promising efficacy in the utility of PARPis for breast cancer treatment. However, the most common adverse events are seemingly related to pancytopenia and associated symptoms which could potentially hinder patient preference and adherence. As such, whilst these trials provide valuable insights into the potential therapeutic benefits of neoadjuvant PARPis, they collectively highlight the need for further research to optimize and enhance treatment strategies.

### Ongoing Clinical Trials Evaluating Neoadjuvant PARPis in HRD Cancers

#### Breast Cancer

Trials investigating the use of PARPis in the neoadjuvant setting for breast cancer are aiming to optimize patient selection and combination treatments while minimising toxicity (Table 3). Many current clinical trials are being conducted based on the promising findings from initial pilot studies. The OlympiaN trial is evaluating pCR and event-free survival post neoadjuvant olaparib monotherapy versus in combination with durvalumab for high and low-risk patients with ER-negative/ER-low/HER2-negative breast cancer [65, 66]. This trial also seeks to refine treatment strategies by tailoring therapy based on risk stratification, with the potential to de-escalate traditional chemotherapy in favour of targeted and immune-based approaches [65]. The PARTNER trial investigates if the addition of olaparib to combination paclitaxel and carboplatin neoadjuvant chemotherapy enhances the likelihood of achieving pCR in invasive TNBC or gBRCAm, HER2-negative with any progesterone/estrogen receptor (PgR/ER) status following neoadjuvant treatment and following breast ± axillary surgery [62, 67]. This study seeks to refine treatment strategies by investigating molecular markers and offer novel insights into optimizing neoadjuvant approaches for this high-risk population [67].The PHOENIX trial has combined olaparib, AZD6738, and durvalumab for treatment of post-NACT high residual stage II-III TNBC [68]. The end goal of this trial is analysing changes in gene proliferation measured by tumour cell Ki67 immunohistochemistry to generate insights into the tumor microenvironment and identify biomarkers that may guide future therapeutic strategies for chemotherapy-resistant disease [68]. The COGNITION-GUIDE trial investigates a combination of treatments—including atezolizumab, inavolisib, ipatasertib, olaparib, sacituzumab govitecan, trastuzumab, and pertuzumab—for early-stage (I-III) high risk TNBC or HER2-positive breast cancer [69]. The trial's primary goal is to evaluate invasive disease-free survival [69, 70]. It also aims to tailor treatment based on the molecular characterization of tumors, with olaparib specifically designated for patients with somatic or germline BRCA mutations or inactivating germline PALB2 mutations [69].

**Table 3:** Summary of ongoing trials investigating PARPis in the neoadjuvant setting for breast cancer

Study	Year	Phase	Disease stage	Inclusion Criteria	Neoadjuvant treatment	Treatment Regimen	Control	Endpoints
OlympiaN (NCT05498155)	2022	II	All	HER2-, *BRCA*m	Olaparib + Durvalumab	Olaparib PO 300 mg × 2 BID or combination with 1500 mg Durvalumab IV infusion q4week for 4 –6 × 28-day cycles		pCR, RCB
(NCT05834582)	2023	II	II-III	*BRCA*1/2, HER2-	Fluzoparib + Paclitaxel or Epirubicin + Cyclophosphamide (EC)	Fluzoparib + Paclitaxel for 4 cycles if tumor response is SD after 2 cycles of EC induced chemotherapy		TpCR, EFS
PARTNER (NCT03150576)	2024	II/III	All	TNBC and/or g*BRCAm*	Paclitaxel + Carboplatin and Olaparib	Paclitaxel I.V. 80 mg/m2 in 0.9% sodium chloride 500 ml over 60 min on d1, 8 & 15, q3week for 4 cycles Carboplatin I.V. in 5% dextrose 500 ml over 30–60 min on day 1 q3weeks for 4 cycles. Self-administer Olaparib by mouth BID 12 h apart	Paclitaxel + Carboplatin only	pCR
NCT04481113	2020	I	All	Positive (HR +) HER2-	Abemaciclib, Niraparib Tosylate Monohydrate	Abemaciclib PO BID and niraparib PO QD. Treatment repeats every 28 days for up to 2–4 cycles in the absence of disease progression or unacceptable toxicity		ORR, CBR, pCR, RCB
IMPARP (NCT05761470)	2023	II	I-II	HER2-, HRR gene mutation	Camrelizumab, Fluzoparib and Nab-paclitaxel	Camrelizumab 200 mg IV infusion each 21 day cycle. Fluzoparibat 100 mg BID each 21 day cycle. Nab-paclitaxel 260 mg (63) infusion on d1 each 21-day cycle		pCR
COGNITION-GUIDE (NCT05332561)	2023	II	I-III	Early (stage I-III) TNBC or HER2-positive/negative breast cancer,	Atezolizumab, Inavolisib, Ipatasertib, Olaparib, Sacituzumab Govitecan, Trastuzumab/Pertuzumab	Atezolizumab 1200 mg IV, on d1, q21d; Inavolisib 9 mg PO, on d1-d28, q28d; Ipatasertib Dosage: 400 mg, PO, on d1-d21, q28d; Olaparib 300 mg, PO, b.i.d d1-d28, q28d; Sacituzumab Govitecan 10 mg/kg BW, IV, on d1 and d8, q21d; Trastuzumab/Pertuzumab SC; Initial dose: Trastuzumab 600 mg, Pertuzumab 1200 mg, 30 000 units hyaluronidase; Maintenance dose: Trastuzumab 600 mg, Pertuzumab 600 mg, 20 000 units hyaluronidase; Frequency: on d1, q21d		DFS
PHOENIX (NCT03740893)	2020	II	II-III	NACT resistant residual TNBC	Olaparib, AZD6738, Durvalumab	Pre-operative exposure of 160 mg AZD6738 PO BID on d 5 −14 of the WOP. Pre-operative exposure to 300 mg of Olaparib PO b.i.d. on d1-14 of the WOP. Pre-operative exposure to 1500 mg durvalumab IV infusion on d1 only of the WOP. Cohort Dependant		Gene proliferation profile and index

Abbreviations: pCR = pathological complete response, ORR = overall response rate, TRS = tumour resection success, EFS = Event-free Survival, CBR = Clinical benefit rate, RCB = Rate of residual cancer burden, WOP = window-of-opportunity, DFS = disease-free survival, tpCR = total pathological complete response, TNBC = triple negative breast cancer, SD = stable disease, PO = oral, b.i.d. = twice a day, IV = intravenous, q4week = every 4 weeks, gBRCAm = germline BRCA mutated, HR +  = hormone receptor positive, HER2- = human epidermal growth factor receptor 2 negative, HRR = homologous recombination repair, SC = subcutaneous, BW = body weight, NACT = neoadjuvant chemotherapy, AZD6738 = a specific investigational drug.

A newer PARPi currently being utilized within these ongoing trials for breast cancer is fluzoparib. The IMPARP trial investigates the combination of fluzoparib, camrelizumab and nanoparticle albumin-bound (nab) paclitaxel in HER2-negative HRD BC, with pCR as the measured endpoint [71]. Fluzoparib is also being investigated in trial NCT05834582, exploring its implementation and combination of with paclitaxel following 2–4 cycles of epirubicin/ cyclophosphamide in advanced breast cancer in stages II-III with pCR being the main endpoint and the secondary end point of EFS [72]. Finally, an NCT04481113 is examining the combination of the treatments abemaciclib, niraparib tosylate monohydrate with the primary end goal of focusing on incidence of dose limiting toxicities (DLTs) and AEs [73]. The results of these ongoing trials may increase clinicians’ understanding of the impacts of diverse implementations of PARP inhibitors in the neoadjuvant setting for breast cancer, allowing for increased ability to tailor medication regiments in the future and best select patients for their use.

### Ovarian Cancer

Although most research efforts have been focused on utilizing PARPis in a neoadjuvant setting for breast cancer, emerging studies are investigating PARPis in the setting of ovarian cancer (Table 4). Currently, the phase I NOW trial is assessing neoadjuvant olaparib in advanced ovarian, peritoneal, or fallopian tube carcinoma [74]. Pilot results report a 100% tumour resection success (TRS), 86% complete gross resection, and 8% pCR, justifying further investigations into the efficacy of olaparib neoadjuvant monotherapy [74]. Previous trials have highlighted the efficacy of PARPis, such as niraparib, in managing recurrent ovarian cancer, and ongoing research continues to investigate the potential of other PARPi treatments in this setting [75].

**Table 4:** Summary of trials investigating PARPis in the neoadjuvant setting for ovarian cancer

Study (trial number)	Year	Phase	Study population	Inclusion Criteria	Neoadjuvant treatment	Treatment Regimen	Control	Endpoints	Safety and Toxicity (if reported)
NOW (NCT03943173)	2024	I	Advanced ovarian, peritoneal, or fallopian tube carcinoma	*BRCA* mutations	PARPi olaparib (n = 15)	Olaparib PO b.i.d on days 1–28 repeats every 28 days for up to 2 cycles		100% TRS, 86% complete gross resection, 8% pCR	
NANT (NCT04507841)	2022	II	Stages III-IV advanced ovarian, peritoneal, or fallopian tube cancers	g*BRCA*1/2 OR HRD	PARPi niraparib	Niraparib 100 mg PO BID		ORR	
AMBITION (NCT03699449)	2021	II	Platinum-resistant OC	HRD	PARPi olaparib + cediranib OR durvalumab (n = 16, 14)	Cediranib 30 mg PO QD and olaparib 200 mg PO b.i.d OR durvalumab 1500 mg IV every 4 weeks starting on week 5 day1 for up to 12 months and olaparib 300 mg PO b.i.d until disease progression		ORR 50%, 42.9%	≥ 3 AE: 37.5% cediranib and 35.7% durvalumab
NUVOLA (NCT04261465)	2020	II	Stages III-IV primary ovarian, peritoneal, or fallopian tube cancers	g*BRCA*1/2	carboplatin + paclitaxel + intermittent olaparib (n = 35)	Paclitaxel 60 mg/m2 IV and carboplatin AUC 2 IV for 3 weeks out of 4, and olaparib 150 mg PO b.i.d for 3 consecutive days every week for 3 cycles		pCR	
OPAL (NCT03574779)	2022	II	Stage III/IV OC	HRD	PARPi niraparib	Niraparib 200 or 300 mg for 3 21-day cycles (n = 1)	Platinum-taxane doublet CT	Pre-interval debulking surgery ORR	≥ 3 AE: 100%
BrUOG 390 (NCT04598321)	2023	I	gBRCA advanced OC	g*BRCA*1/2	PARPi talazoparib	Talazoparib 1 mg PO QD for 21 days		Preliminary Effectiveness	

Abbreviations: OC = ovarian cancer, pCR = pathological complete response, ORR = overall response rate, TRS = tumour resection success, gBRCA = germline BRCA, PO = oral, QD = once daily, AE = adverse event, PARPi = poly(ADP-ribose) polymerase inhibitor, b.i.d = twice a day, HRD = homologous recombination deficiency, IV = intravenous, AUC = area under curve, CT = chemotherapy.

The AMBITION trial, phase II, targets platinum-resistant ovarian cancer, evaluating olaparib combined with cediranib or durvalumab, yielding ORR of 50% and 42.9%, respectively [76, 77]. The NUVOLA trial assesses a regimen of carboplatin, paclitaxel, with intermittent olaparib in advanced OC, with pCR as the primary endpoint [76]. The OPAL compares niraparib to platinum-taxane doublet chemotherapy, focusing on pre-interval debulking surgery ORR as the primary endpoint [78]. Lastly, the NANT trial studies neoadjuvant niraparib in stage III-IV advanced ovarian, peritoneal, or fallopian tube cancers, with ORR as the endpoint [76]. Results from the ongoing trials will inform us of the efficacy of PARPis for the neoadjuvant treatment of ovarian cancer. The BrUOG 390 trial, a phase I trial, initiated in 2023 and then terminated due to financial termination of research grant agreement, with no additional details provided regarding recruitment, safety, or efficacy concerns, investigated talazoparib, evaluating safety and preliminary effectiveness [76]. In this trial, talazoparib was administered as a neoadjuvant treatment at a dose of 1 mg orally once daily (QD) for 21 days [79]. These findings highlight the potential toxicity associated with talazoparib, which may require further testing with different combination therapies or dose-adjustments. As preliminary effectiveness was not reported in this study, future prospects with this PARPi remain unclear.

### Prostate Cancer

There are multiple clinical trials underway assessing neoadjuvant PARPis in different prostate cancer cohorts (Table 5). Currently, the NePtune trial involves treatment with olaparib and LHRH, focusing on pCR as the primary endpoint and MRD as a secondary endpoint [80]. The NCT04030559 trial examines the effects of niraparib in localized PCa, aiming to assess pRR, PSA levels, and PFS [81, 82]. The outcomes of these emerging trials will provide insight into the effectiveness of PARP inhibitors in the neoadjuvant setting for prostate cancer, potentially guiding future therapeutic strategies. The BrUOG337 trial was a phase II trial studying olaparib in patients with localized or advanced PCa, with endpoints measuring prostate specific antigen (PSA) response and progression free survival (PFS) [81, 83]. It was terminated due to a lack of enrolment secondary to eligibility criteria, but published results were based on one participant [84]. Results showed no significant PSA response and a PFS of 7 months [84]. No serious AEs were reported, however the participant did experience systemic AEs such as anaemia, oedema, and fatigue [84]. Additional results from a larger cohort are essential to determine the true effectiveness of olaparib in treating prostate cancer.

**Table 5:** Summary of trials investigating PARPis in the neoadjuvant setting for prostate cancer

Study (trial number)	Year	Phase	Study population	Inclusion Criteria	Neoadjuvant treatment	Treatment Regimen	Control	Endpoints	Safety and Toxicity (if reported)
NePtune (NCT05498272)	2024	II	Localised PCa	g*BRCA*1/2	PARPi olaparib and LHRH	180 days: olaparib 300 mg PO BID 6 Cycles (30 day Cycles); LNRH agonist		pCR + MRD	
Niraparib Before Surgery (NCT04030559)	2024	II	Localised PCa	HRD: defects in genes *BRCA1/2, ATM, CHEK1/2 FANCA, FANCD2, FANCL, GEN1, NBN, PALB2, RAD51, RAD51c*, and *BRIP1*	Niraparib (estimated n = 30)	Niraparib PO QD on days 1–28 up to 3 cycles		pRR + PSA PFS	
BrUOG337 (NCT03432897)	2022	II	Localised or advanced PCa	Defects in genes *BRCA1, BRCA 2, ATM, CHEK1, CHEK2, FANCL*, *HDAC2, PALB2, BARD1, BRIP1, CDK12, PPP2R2A, RAD51B, RAD51C, RAD51D*, or *RAD54L*	Olaparib (estimated n = 13)	Olaparib 300 mg PO BID for 4 weeks for up to 3 cycles		Reduced PSA response + PFS	

Abbreviations: pCR = pathological complete response, PSA = prostate specific antigen, PFS = progression free survival, pRR = pathologic response rate, MRD = minimal residual disease, LHRH = luteinizing hormone-releasing hormone, BID = twice daily, QD = once daily, PO = oral, PCa = prostate cancer, gBRCA1/2 = germline BRCA1/2, PARPi = poly(ADP-ribose) polymerase inhibitor, HRD = homologous recombination deficiency, HDAC2 = histone deacetylase 2, BARD1 = BRCA1-associated ring domain 1, CDK12 = cyclin-dependent kinase 12, PPP2R2A = protein phosphatase 2 regulatory subunit Balpha, RAD51B, RAD51C, RAD51D = Rad51 family of recombinase genes, FANCL = fanconi anemia complementation group L, BRIP1 = BRCA1 interacting protein 1, FANCA, FANCD2, FANCL, GEN1, NBN, PALB2 = genes involved in Fanconi anemia and homologous recombination repair.

### Pancreatic Cancer

Although pancreatic cancer is typically very aggressive and often diagnosed in advanced stages, there remains a gap in the literature on whether PARPis could be efficacious in a neoadjuvant setting and therefore may be underrepresented compared to other HRD-related cancers [85]. For instance, a phase I study (NCT04425876) is currently investigating fuzuloparib in addition to standard of care mFOLFIRINOX as neoadjuvant treatment of pancreatic adenocarcinoma [86]. Patients received up to 12 cycles of mFOLFIRINOX [14 days each], with 4 to 6 cycles administered preoperatively, and escalating doses of fuzuloparib starting at 30 mg twice daily, followed by maintenance fuzuloparib at 150 mg BID [86]. As of October 2023, 3 patients were enrolled in the 30 mg cohort and 12 in the 60 mg cohort [86]. 80.0% of patients completed the neoadjuvant therapy as planned [86]. 53.3% underwent surgical resection, and all achieved R0 resections (95% CI, 63.1–100.0) [86]. Grade ≥ 3 AEs occurred in 80.0% of patients, with the most common being neutropenia, leukopenia, hypokalaemia, and anaemia [86].

## Future Outlooks

Future research in neoadjuvant PARPi therapy should also address several areas to optimize efficacy and clinical application. Identifying reliable biomarkers beyond *BRCA1/2* mutations, (e.g.*ATM*, *PALB2*, and *RAD51* mutations) may improve patient selection [87, 88]. In the neoadjuvant setting, reliable biomarkers could predict response to therapy, improving outcomes by patients who are likely to achieve significant tumour shrinkage or pathologic response before surgery to be selected [87, 88]. Tumours with decreased expression of growth factor receptor-bound protein 2 (GRB2) reflected the same response to PARPis as BRCAm cancers [89]. GRB2 is responsible for stabilizing RAD51 filaments at replication forks, preventing MRE11-mediated degradation, and modulating the cGAS/STING pathway, enhancing PARPi sensitivity and replication stress response [89]. Tumour cells with increased expression of gasdermin C (GSDMC) predicted better treatment response to PARPi in TNBC through the activation of GSDMC/caspase-8–mediated cancer cell pyroptosis (CCP) enhancing tumour microenvironment cytotoxicity [90]. More clinical trials should focus on increasing cohorts to demonstrate meaningful outcomes with neoadjuvant PARPi in prostate and pancreatic cancer.

Additional studies may also further assess combination strategies to enhance therapeutic outcomes and provide alternate treatment regimens. One of the current promising combinations is PARPis with immune checkpoint inhibitors (ICIs) [91, 92]. PARPi–ICI combinations may work by increasing the tumour’s neoantigen load, thereby enhancing immune recognition and response [93]. The MEDIOLA trial explored the combination of olaparib with durvalumab in the treatment of gBRCAm mBC [94]. The trial showed enhanced antitumor activity, with a PFS of 8.2 months compared to olaparib alone (PFS 7.0 months) [94]. Beyond HRD tumours, this process increases the presence of tumour neoantigens, upregulates interferons and PD-L1, and alters the tumour microenvironment enhancing the immune system's ability to attack the cancer [95, 96]. Several trials described above are currently assessing this synergistic therapeutic combination including; the I-SPY2, OlympiaN, PHOENIX, and AMBITION trials [94].

Further research into these combinations could help identify patient subgroups most likely to benefit and explore the potential for combining PARPis with other novel agents, such as anti-angiogenic drugs. By expanding the therapeutic options available, these strategies could significantly improve outcomes for a broader range of cancer patients, particularly those with limited treatment options.

A common, well-reported challenge with PARPi therapy in the adjuvant setting is treatment resistance [97]. While yet to be explicitly reported in the neoadjuvant setting, current AEs and lack of efficacy reported in ongoing trials suggest that resistance mechanisms are likely similarly influencing clinical outcomes. Drug resistance in the neoadjuvant setting is well documented, notably in patients with HER2-positive BC, treated with trastuzumab [98]. In the adjuvant PARPi setting, reversing resistance has become a focus of both in vitro and in vivo studies. Several mechanisms can contribute to PARPi resistance. [99]. One well-documented mechanism of resistance is the restoration of HRR through secondary mutations in *BRCA1/2* genes [100]. Recent research suggests that DNA end resection, tightly under the influence of cyclin-dependent kinases (CDKs), contributes to PARPi resistance by inducing HRR and evading synthetic lethality [101]. Additional mechanisms include mutations in other HR-related genes, such as RAD51C and RAD51D, and the upregulation of drug efflux pumps (e.g., increased expression of the ABCB1 gene) [100, 102]. Additionally, alterations in PARP itself, such as mutations that prevent PARP trapping or changes in post-translational modifications, can also lead to resistance [103]. These mutations can reduce the ability of PARPis to effectively inhibit PARP, thereby allowing cancer cells to survive despite treatment​ (103). Current evidence proposes the combination of PARPis and CDKs inhibitors to overcome CDK mediated resistance [101]. Future research should explore other resistance mechanisms in the neoadjuvant setting.

Furthermore, additional studies are needed to understand long-term efficacy, overall survival, and quality of life impacts of PARPis [104]. One effective way of measuring quality of life impacts during clinical trials is through patient reported outcomes, as it allows a holistic and varied assessment of the treatment regimen [105]. This approach can capture a wide range of effects, including physical, emotional, and social well-being, that might not be fully reflected through clinical or laboratory measures alone [105]. In a study examining patient-reported outcomes (PROs) in a real-world, multinational population of patients with gBRCA1/2 m HER2-negative advanced breast cancer, PARPis were associated with an improved quality of life (QoL) across multiple dimensions compared to chemotherapy. Patients receiving PARPis reported better physical and social functioning, as well as fewer systemic therapy side effects, though nausea and vomiting were more common with PARPis than chemotherapy. Overall, PROs suggest that PARPis are at least as satisfying as chemotherapy, with patients experiencing better health-related QoL [106]. Similarly, adjuvant PARPi trials in ovarian cancer, namely PAOLA-1 and SOLO1, reported no change or an increase in QOL measures with PARPis compared to monotherapy [107]. However, most studies have analysed the effect of PARPis in the advanced setting with further evidence needed to evaluate patients’ satisfaction in the neoadjuvant setting, particularly if their use is to be expanded to those with smaller HRD tumours.

## Conclusion

PARPi show promise as neoadjuvant agents in HRD-positive cancers. In breast cancer, improved pCR rates were observed in some trials, though toxicity remains a concern. Early ovarian cancer studies report encouraging resection rates and ORRs, while prostate and pancreatic cancer data are limited, and more research is needed before drawing strong conclusions on the efficacy of neoadjuvant PARPis. Future trials should focus on biomarker-driven patient selection, combination strategies, and long-term outcomes to fully define the role of neoadjuvant PARPi therapy.

In tumour cells, administering PARPis prevents DNA repair, and causes accumulation of the DNA damage, resulting in cell death. This approach is termed “synthetic lethality” and is utilised as an effective adjuvant treatment for cancer cells with germline mutations in HRR pathway. Abbreviations: HRR, Homologous Recombination Repair; DNA, deoxyribonucleic acid. Created from https://www.biorender.com/.

Patient diagnosed with cancer with high risk of germline mutation. Patient undergoes genetic counselling and testing for germline mutations in HRR genes and further assays to assess degree of genomic instability and confirm HRD. Once a patient is confirmed to have HRD, they undergo PARPi therapy to provide prognostic information and make surgical resection more manageable. Abbreviations: HRD = Homologous Repair Deficiency; BRCA1/2; ATM. Created from https://www.biorender.com/.

## Key References

- Geyer CE, Jr., Garber JE, Gelber RD, Yothers G, Taboada M, Ross L, et al. Overall survival in the OlympiA phase III trial of adjuvant olaparib in patients with germline pathogenic variants in BRCA1/2 and high-risk, early breast cancer. Ann Oncol. 2022;33(12):1250–68.
  o One of the first trials that gained licencing for olaparib in the adjuvant setting.
- Pusztai L, Yau C, Wolf DM, Han HS, Du L, Wallace AM, et al. Durvalumab with olaparib and paclitaxel for high-risk HER2-negative stage II/III breast cancer: Results from the adaptively randomized I-SPY2 trial. Cancer Cell. 2021;39(7):989–98.e5.
  o One of the first trials to demonstrate the efficacy of olaparib in the neoadjuvant setting.
- Yu Y, Zhang W, Liu R, Shan W, Li H, Liu J, et al. Effectiveness and safety of niraparib as neoadjuvant therapy in advanced ovarian cancer with homologous recombination deficiency: NANT study protocol for a prospective, multicenter, exploratory, phase 2, single-arm study (041). Gynecologic Oncology. 2022;166:S28-S9.
  o This study, currently active, is the first prospective study to investigate the benefit and safety of niraparib in neoadjuvant treatment for advanced OC.
- Zhan Q, Wen C, Zou S, Li F, Chen D, Sheng Z, et al. Perioperative fuzuloparib plus mFOLFIRINOX for resectable pancreatic adenocarcinoma: A phase 1 study. Journal of Clinical Oncology. 2024;42(16_suppl):4163-.
  o This is one of the only studies that explores neoadjuvant PARPi in the treatment of pancreatic cancer. The addition of neoadjuvant fuzuloparib to current treatment standard demonstrates R0 resection rate of 100%.
- Fasching PA, Link T, Hauke J, Seither F, Jackisch C, Klare P, et al. Neoadjuvant paclitaxel/olaparib in comparison to paclitaxel/carboplatinum in patients with HER2-negative breast cancer and homologous recombination deficiency (GeparOLA study). Ann Oncol. 2021;32(1):49–57.
  o This study demonstrates that the addition of targeted PARPi therapy to paclitaxel in the neoadjuvant setting is les​s toxic than dual chemotherapy, in patients with HRD/TNBC.
- Litton JK, Beck JT, Jones JM, Andersen J, Blum JL, Mina LA, et al. Neoadjuvant Talazoparib in Patients With Germline BRCA1/2 Mutation-Positive, Early-Stage Triple-Negative Breast Cancer: Results of a Phase II Study. Oncologist. 2023;28(10):845–55.
  o This trial demonstrated that neoadjuvant talazoparib therapy was well tolerated.
- Li N, Zhu J, Yin R, Wang J, Pan L, Kong B, et al. Treatment with niraparib maintenance therapy in patients with newly diagnosed advanced ovarian cancer: a phase 3 randomized clinical trial. JAMA oncology. 2023;9(9):1230–7.
  o This study investigates the efficacy of niraparib in multiple populations and tumor statuses with ovarian cancer.
- Banerjee S, Moore KN, Colombo N, Scambia G, Kim B-G, Oaknin A, et al. Maintenance olaparib for patients with newly diagnosed advanced ovarian cancer and a BRCA mutation (SOLO1/GOG 3004): 5-year follow-up of a randomised, double-blind, placebo-controlled, phase 3 trial. The Lancet Oncology. 2021;22(12):1721–31.
  o This study demonstrates promising 5-year PFS of 56.0 months with olaparib monotherapy compared to 13.8 months with placebo. 260 patients were randomly allocated to olaparib monotherapy and 131 to placebo.
- Ray-Coquard I, Leary A, Pignata S, Cropet C, González-Martín A, Marth C, et al. Olaparib plus bevacizumab first-line maintenance in ovarian cancer: final overall survival results from the PAOLA-1/ENGOT-ov25 trial. Annals of Oncology. 2023;34(8):681–92.
  o This phase III trial demonstrated significant benefit of the addition of olaparib to bevacizumab compared to placebo plus bevacizumab, demonstrating therapeutic benefit of combining PARPis with other therapies.
- Clarke NW, Armstrong AJ, Thiery-Vuillemin A, Oya M, Shore N, Loredo E, et al. Abiraterone and Olaparib for Metastatic Castration-Resistant Prostate Cancer. NEJM Evidence. 2022;1(9):EVIDoa2200043.
  o This study explored the combination of androgen inhibition with PARPi in mCRPC, showing improved outcomes regardless of HRR status.
- de Bono JS, Mehra N, Scagliotti GV, Castro E, Dorff T, Stirling A, et al. Talazoparib monotherapy in metastatic castration-resistant prostate cancer with DNA repair alterations (TALAPRO-1): an open-label, phase 2 trial. Lancet Oncol. 2021;22(9):1250–64.
  o Results of this study support future large-scale studies of talazoparib in mCRPC.
- Abida W, Campbell D, Patnaik A, Bryce AH, Shapiro J, Bambury RM, et al. Rucaparib for the Treatment of Metastatic Castration-resistant Prostate Cancer Associated with a DNA Damage Repair Gene Alteration: Final Results from the Phase 2 TRITON2 Study. Eur Urol. 2023;84(3):321–30.
  o This study enrolled 277 patients, with different gene mutated mCRPC, in which half of patients improved on rucaparib therapy.
- Spring LM, Han H, Liu MC, Hamilton E, Irie H, Santa-Maria CA, et al. Neoadjuvant study of niraparib in patients with HER2-negative, BRCA-mutated, resectable breast cancer. Nat Cancer. 2022;3(8):927–31.
  o This trial demonstrated the therapeutic benefit of niraparib in localized HRD BC.
- Westin S, Michael V, Fellman B, Meyer L, Taylor T, Alvarado T, et al. Now: neoadjuvant olaparib window trial in patients with newly diagnosed BRCA mutant ovarian cancer (LBA 1). Gynecologic Oncology. 2023;176:S2-S3.
  o This study investigates the use of olaparib in neodjuvant treatment of ovarian cancer with pilot results demonstrating promising results.
- Dall'era M, McPherson J, Evans CP, Parikh M, Verma R, Lara PN. Neo-adjuvant PARP inhibition prior to prostatectomy: A phase II biomarker-driven trial for men with high-risk, localized prostate cancer and alterations in DNA repair genes. American Society of Clinical Oncology; 2023.
  o Currently recruiting, this study aims to evaluate pCR at prostatectomy following neoadjuvant niraprib.

## Data Availability

No datasets were generated or analysed during the current study.
